# Hypochlorous acid inactivates oral pathogens and a SARS-CoV-2-surrogate

**DOI:** 10.1186/s12903-023-02820-7

**Published:** 2023-02-18

**Authors:** Kento Tazawa, Rutuja Jadhav, Mariane Maffei Azuma, J. Christopher Fenno, Neville J. McDonald, Hajime Sasaki

**Affiliations:** 1 Department of Cariology, Restorative Sciences, and Endodontics, School of Dentistry, University of Michigan, 1011 N University Ave, Ann Arbor, MI 48109 USA; 2 Division of Oral Health Sciences, Department of Pulp Biology and Endodontics, Graduate School of Medical and Dental Sciences, Tokyo Medical and Dental University (TMDU), Tokyo, Japan; 3 Department of Biologic and Materials Sciences & Prosthodontics, School of Dentistry, University of Michigan, Ann Arbor, MI 48109 USA

**Keywords:** Hypochlorous acid (HOCl), Dental practice, Airborne infection, Oral pathogens, SARS-CoV-2, Disinfectant, Mouthwash, Minimum inhibitory volume ratio

## Abstract

**Background:**

Droplets and aerosols produced during dental procedures are a risk factor for microbial and viral transmission. Unlike sodium hypochlorite, hypochlorous acid (HOCl) is nontoxic to tissues but still exhibits broad microbicidal effect. HOCl solution may be applicable as a supplement to water and/or mouthwash. This study aims to evaluate the effectiveness of HOCl solution on common human oral pathogens and a SARS-CoV-2 surrogate MHV A59 virus, considering the dental practice environment.

**Methods:**

HOCl was generated by electrolysis of 3% hydrochloric acid. The effect of HOCl on human oral pathogens, *Fusobacterium nucleatum, Prevotella intermedia, Streptococcus intermedius, Parvimonas micra,* and MHV A59 virus was studied from four perspectives: concentration; volume; presence of saliva; and storage. HOCl solution in different conditions was utilized in bactericidal and virucidal assays, and the minimum inhibitory volume ratio that is required to completely inhibit the pathogens was determined.

**Results:**

In the absence of saliva, the minimum inhibitory volume ratio of freshly prepared HOCl solution (45–60 ppm) was 4:1 for bacterial suspensions and 6:1 for viral suspensions. The presence of saliva increased the minimum inhibitory volume ratio to 8:1 and 7:1 for bacteria and viruses, respectively. Applying a higher concentration of HOCl solution (220 or 330 ppm) did not lead to a significant decrease in the minimum inhibitory volume ratio against *S. intermedius* and *P. micra*. The minimum inhibitory volume ratio increases in applications of HOCl solution via the dental unit water line. One week of storage of HOCl solution degraded HOCl and increased the minimum growth inhibition volume ratio.

**Conclusions:**

HOCl solution (45–60 ppm) is still effective against oral pathogens and SAR-CoV-2 surrogate viruses even in the presence of saliva and after passing through the dental unit water line. This study indicates that the HOCl solution can be used as therapeutic water or mouthwash and may ultimately reduce the risk of airborne infection in dental practice.

## Introduction

Airborne infection is a long-standing problem yet to be solved in dental practice. The coronavirus disease of 2019 (COVID-19) pandemic underscores the importance of this issue and has increased interest in environmental disinfection measures. In the oral cavity, pathogens harbor in various sites, including saliva, mucous membranes, and tooth surfaces [1–3]. The oral cavity is also a harbor of severe acute respiratory syndrome coronavirus 2 (SARS-CoV-2), which causes COVID-19, and viral particles have been detected in the saliva of COVID-19 patients [4, 5]. Most routine dental procedures generate aerosols from the use of rotary instruments, air syringe, or ultrasonic scaler, forced air, sonication, and water derived from contaminated dental unit water line (DUWL). These operations routinely produce a profusion of contaminated particles in a range of sizes that include both droplets (a size larger than 5 µm) and aerosols (a size smaller than 5 µm) [6], which are released into the air and spread pathogens [1, 2, 7, 8]. Indeed, 16 bacterial and 23 fungal species have been found in bio-aerosols in the dental environment [1]; the oral-derived aerosol is considered as a major route of infection transmission between patients and dental care providers [9]. In this context, several factors affect the risk of airborne infection, including such as the proximity to the source and the duration of exposure; combining these factors increases the risk [10]. The emission of contaminated particles is denser the closer they are to the source, the oral cavity. Aerosols are smaller/lighter than droplets, allowing them to remain airborne for extended periods of time, increasing the risk of infection [11, 12]. Sustained proximity to a patient during aerosol generating procedures exposes care providers to dense contaminated emissions and faces a high risk for infection. Furthermore, in poorly ventilated spaces, contaminated aerosols can accumulate, leading to a greater risk for infection [10, 13]. Personal protective equipment such as N-95 masks and environmental controls including dental dam, saliva ejector, high-volume evacuator, and general ventilation are risk mitigating factors reducing exposure to contaminated aerosols; however, dental health care providers are not fully protected from the risk by these measures as the spread of aerosols cannot be completely blocked [13, 14]. Therefore, inactivation of the pathogen prior to aerosol spread would be critical for effective prevention of airborne infection transmission.

Hypochlorous acid (HOCl) is a powerful oxidant that exerts its broad-spectrum microbicidal effect through inhibition of Adenosine 5ʹ-triphosphate synthesis, structure and replication of nucleic acids, protein synthesis, and cell wall synthesis [15–17]. To date, there have been numerous reports on the potent microbicidal activity and safety of HOCl in environmental disinfection and antisepsis [18–20]. In addition, the efficacy of topically applied HOCl in promoting wound healing and its mechanism of action has been reported [21–23]. Based on this evidence, HOCl solution is included in the World Health Organization (WHO) list of coronavirus-effective biocides and the US Environmental Protection Agency ‘N’ list of disinfecting agents able to control emerging pathogens, including SARS-CoV-2 [24, 25]. The US Food and Drug Administration has cleared aqueous HOCl formulations for topical use in wound management. In addition, inexpensive but reliable generators of HOCl solutions are commercially available. An ideal disinfectant should be nontoxic to humans, effective against a wide range of pathogens, and relatively inexpensive; HOCl meets all these requirements for disinfectants.

Taking the advantage of HOCl above, reliable decontamination of treatment-derived aerosols before their diffusion is an advanced and revolutionary concept that has never been achieved in dental infection control. To date, potential clinical applications of HOCl in the field of dentistry, such as mouthwash and water treatment of dental units, have been discussed [26, 27]. However, there is a lack of systematic basic data showing the efficacy of HOCl against oral pathogens. The present study aimed to evaluate the efficacy of HOCl against human oral pathogens and a SARS-CoV-2 surrogate, considering factors such as concentration, amount, time of application, presence of saliva, and DUWLs.

## Materials and methods

### Preparation of hypochlorous acid and microorganisms

Hypochlorous acid was generated by electrolysis of 3% hydrochloric acid using an Apia Mini generator (Hokuty Hokuetsu Co. Ltd., Kanagawa, Japan). Additional electrolysis cycles applied to obtain higher concentration HOCl solutions. HOCl solutions with 45–60 ppm total chlorine content were used in all assays unless otherwise specified. The total chlorine content was determined using the UHR chlorine photometer (HI96734) and Chlorine Ultra High Range Reagent Set (HI95771-01; both Hanna Instruments, Inc., Woonsocket, RI, USA). Stored-HOCl was prepared by placing fresh HOCl solution in a plastic bottle and allowing it to stand at room temperature for 1 week. The average total chlorine concentration of stored-HOCl was 26 ppm. HOCl solution passed through DUWL in use (DUWL-HOCl) was obtained at the University of Michigan Graduate Endodontics Clinic as follows. The operating dental unit water was replaced with HOCl solution in dental chairs at the Graduate Endodontics Clinic. After flushing the residual regular water out using a sterilized 3-in-1 syringe, HOCl sample dispensed from DUWL was collected and immediately used. Note that the dental chairs are regularly treated with Citrisil (daily) and Citrisil All-In-One (once a month; both Sterisil, Inc., Palmer Lake, CO, USA). *Fusobacterium nucleatum* (Fn; American Type Culture Collection (ATCC) 25,586, ATCC, Manassas, VA, USA), *Prevotella intermedia* (Pi; ATCC 25,611), *Streptococcus intermedius* (Si; ATCC 27,335), *Parvimonas micra* (Pm; ATCC 33,270) were grown on tryptone soy agar (TSA) plate with sheep blood (R01202, 5% Sheep Blood in Tryptic soy agar, Remel, Lenexa, KS, USA) in an anaerobic chamber (85% N_2_, 10% H_2_, and 5% CO_2_). Oxyrase (Oxyrase Inc., Mansfield, OH, USA) was used following the manufacturer's instructions in order to reduce the effect of atmospheric oxygen in the suspension preparation. The bacterial suspension was diluted by Oxyrase-PBS solution and adjusted to an optical density (OD) of 1.0 (1.0 × 10^9^ cells/ml) in all bactericidal assays. OD was measured with a spectrophotometer (Thermo Fisher Scientific, Genesys 20, Rochester, NY, USA) with 600 nm wavelength. The bacterial suspensions were freshly prepared on a per-experiment basis.

### Evaluation of HOCl efficacy on oral pathogens in vitro

The impact of usage, storage, residual chlorine content, organic matter, and supply route on the microbicidal activity of HOCl solution was evaluated as a change in the "minimum inhibitory volume ratio," which is how much HOCl solution volume is required to completely inactivate a suspension of pathogens. HOCl solution and bacterial suspensions (OD = 1) were mixed at various volume ratios (Range: 0.25: 1–16: 1 [HOCl: Bacteria by volume]) under ambient air. The mixtures were vortexed, and then chlorine was neutralized with 10 µl of 0.5% Sodium thiosulphate. The entire process took less than 30 s to avoid the influence of ambient oxygen as much as possible. Prepared samples (200 µl of each) were anaerobically cultured and triplicate samples were prepared per condition. Oxyrase-treated PBS (no HOCl solution) served as a control and applied to bacterial suspension in the same protocol.

In the experiment with stored-HOCl, Pi was used as the representative strain. To evaluate the effect of presence of saliva, commercially available human saliva (991-05-P, Lee Biosolutions, Maryland Heights, MO) was employed. Given possible bacteria in the saliva, the saliva was subjected to three freeze–thaw cycles prior to the experiment. After the freeze–thaw cycles, eradication of bacteria was confirmed by anaerobic culture of the saliva on TSA plate with sheep blood for 48 h. The saliva was mixed with Oxyrase, and Oxyrase-saliva was used for microbial suspensions instead of Oxyrase-PBS. The saliva itself used in this study was preliminarily confirmed to have no effect on bacterial growth.

### Preparation of host cells and virus stock

Mouse hepatitis virus (ATCC VR-764 (MHV-A59)) was employed as a SARS-CoV-2 surrogate and propagated in NCTC clone 1469 cells (ATCC CCL-9.1) following ATCC’s instruction. In brief, NCTC clone 1469 cells were cultured in NTCT135 medium (NC1804122, Life Technologies, USA) containing 10% horse serum (H1270, MilliporeSigma, USA) at 37 °C with 5% CO_2_ and 100% relative humidity. Cultured NCTC cells were seeded in 96-well at 3.0 × 10^4^ cells/ well as host cells. MHV-59 virus was diluted 1:100 in serum-free NCTC135 medium and inoculated into the host cells. After 1 h incubation, virus suspension was replaced with NTCT135 medium containing 2% horse serum [28]. The inoculated cells were cultured for 7 days, and the culture supernatants containing the virus were harvested, quickly frozen, and stored as viral stock in liquid nitrogen until use.

**Fig. 1:**
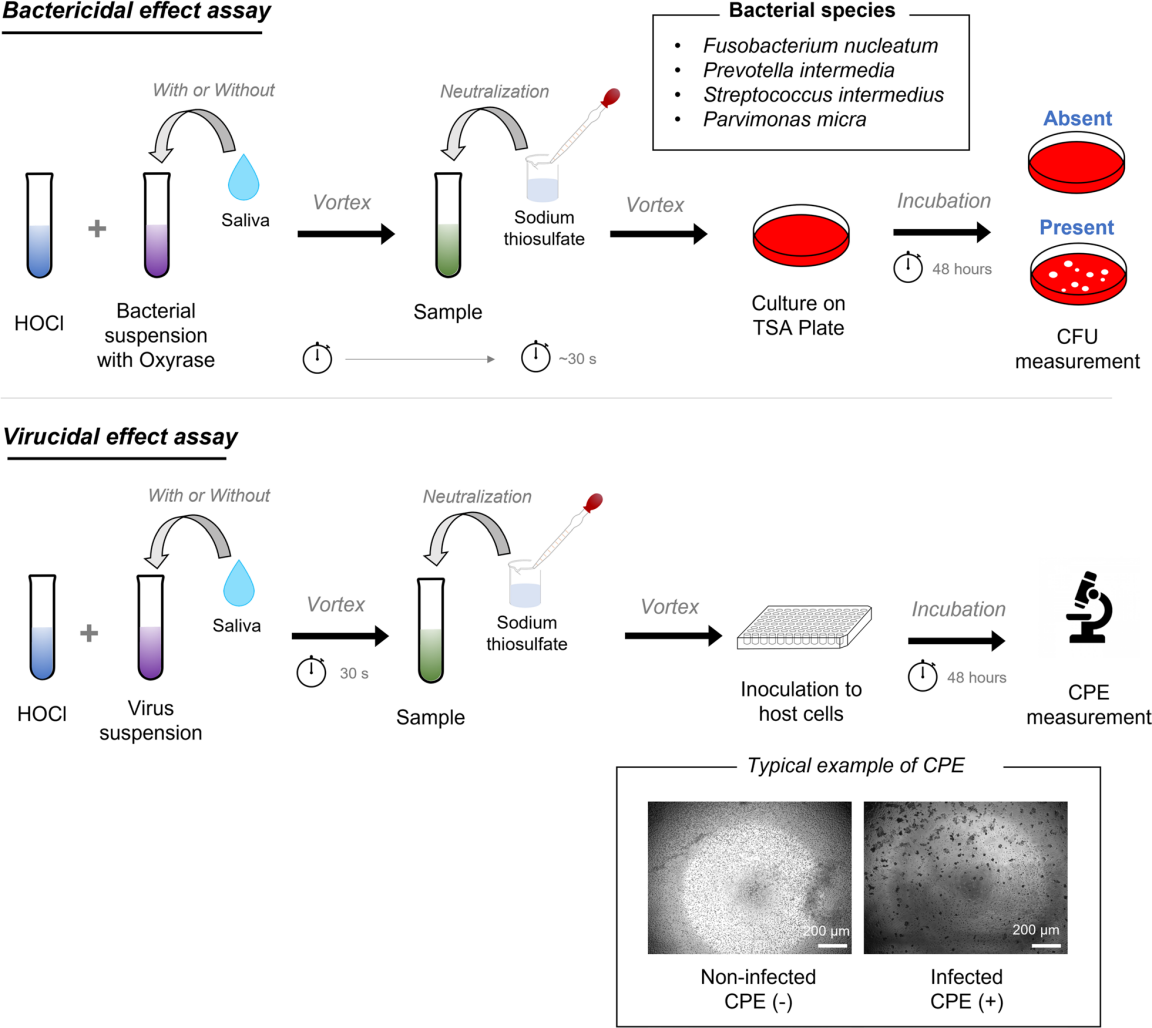
Schematic of the key steps in bactericidal and virucidal assays detailed in Materials and methods

Viral titer was evaluated by endpoint dilution assay using NCTC clone 1469 cells. NTCT clone 1469 cells were seeded in 96-well plates with NCTC135 medium at 3.0 × 10^4^ cells/well containing 10% horse serum and incubated for 24 h at 37 °C. The virus solution was diluted with NTCT 135 medium in serial tenfold dilution and used for inoculation. Cytopathogenic effect (CPE) scores were assessed by inverted phase contrast microscopy on the second day after inoculation. The virus titer (TCID50) was calculated using the method of Reed-Muench [29]. Virus stock solutions with a titer of 6.8 × 10^5^ (TCID 50/ml, in a single lot) were used in all virucidal assays. The procedures of these bactericidal and virucidal assays were summarized in Fig. 1.

### Evaluation of virucidal efficacy of HOCl solution

The virus stock was thawed in a 37 °C water bath. To determine the minimum inhibitory volume ratio for MHV-59 virus, the virus stock was mixed with HOCl solution at the various volume ratios for 30 s at room temperature. Then, effective chloride was neutralized with sodium thiosulfate as described above. The mixtures were diluted 1:100 in NTCT135 medium and inoculated to the host cells. The minimum volume ratio at which CPE was not observed was determined on day 2 after infection as described above. For the effect of saliva, saliva was pre-treated with a protease inhibitor (Halt^™^ protease inhibitor #1862209, Thermo Scientific) for 1 h at room temperature to reduce the negative effect of the protease on host cells. The volume ratio of saliva to virus suspension was pre-optimized to 9:1 because excess saliva inhibits the viral infection to host cells. A saliva-virus suspension was incubated for 10 min at room temperature. The saliva-virus suspension was treated with HOCl at various volume ratios as above. Sample dilution, inoculation, and CPE evaluation are described above.

## Results

First, freshly prepared HOCl solutions (45–60 ppm chlorine content) were applied at various volume ratios to bacterial suspensions (1.0 × 10^9^ cells/ml) in the absence of saliva to determine the minimum inhibition volume ratio (Table 1). The sensitivity of bacteria to HOCl solution tended to vary among the species, with Fn and Pi appearing to be more sensitive compared to Si and Pm. However, at the volume ratio of 4:1 (4 × HOCl solution to bacterial suspension), all species were completely inhibited within 30 s. Thus, the minimum inhibitory volume ratio for common human oral pathogens was 4:1.

**Table 1:** Effect of freshly prepared/stored HOCl solution at various mixing volume ratios on oral pathogens

Bacteria	HOCl	Mixing volume ratio (HOCl: bacterial suspension)						
		0.25:1	0.5:1	1:1	2:1	4:1	8:1	16:1
*F. nucleatum*	Fresh	+	+	−	−	−	−	−
*P. intermedia*	Fresh	+	+	+	−	−	−	−
	Stored	+	+	+	+	−	−	−
*P. micra*	Fresh	+	+	+	+	−	−	−
*S. intermedius*	Fresh	+	+	+	+	−	−	−

+ : Growth was observed. −: No growth was observed

Using Pi as a representative specie, the effect of storage on the minimum inhibitory volume ratio was determined. One week storage of HOCl solution reduced the chlorine content from 46 to 26 ppm; the minimum inhibitory volume ratio of fresh HOCl solution to Pi was 2:1 but increased to 4:1 in the stored solution (Table 1).

Next, we examined whether high concentrations could decrease the minimum inhibitory volume ratio. In this experiment, high concentrations of HOCl solution applied to the bacterial suspension at a 2:1 volume ratio, which is smaller than the minimum inhibitory volume ratio for common oral pathogens above. As shown in Table 2, high concentrations of HOCl were as effective as medium concentrations of HOCl for Fn and Pi (Table 1), but even the highest concentrations did not reduce the minimum inhibitory volume ratio of Si and Pm.

**Table 2:** Bactericidal effect of HOCl solution at high chlorine concentrations

Bacteria	Chlorine concentration (ppm)	
	220	330
*F. nucleatum*	−	−
*P. intermedia*	−	−
*P. micra*	+	+
*S. intermedius*	+	+

The mixing volume of HOCl was double that of the bacterial suspension

+ : Growth was observed. –: No growth was observed

Saliva has a non-negligible effect on oral disinfection. Saliva contains various organic substances, which reduce the bactericidal effect of HOCl. Thus, we examined the extent to which saliva reduced the bactericidal effect of HOCl. In the presence of saliva, the minimum inhibitory volume ratio for common oral pathogens increased to 8:1 from 4:1 (Table 3).

**Table 3:** Bactericidal activity of fresh-HOCl solution in the presence of saliva

Bacteria	HOCl	Mixing volume ratio (HOCl: bacterial suspension)						
		0.25:1	0.5:1	1:1	2:1	4:1	8:1	16:1
*F. nucleatum*	Fresh	+	+	+	+	−	−	−
*P. intermedia*	Fresh	+	+	+	+	+	−	−
*P. micra*	Fresh	+	+	+	+	+	−	−
*S. intermedius*	Fresh	+	+	+	+	+	−	−

+ : Growth was observed. −: No growth was observed

Besides bacteria, the virucidal efficacy of a medium concentration of HOCl solution was examined using MHV-A59, a surrogate virus for SAR-CoV-2. In the absence of saliva, the minimum inhibitory volume ratio for the surrogate virus was 6:1; in the presence of saliva, the minimum inhibitory volume ratio for the surrogate virus increased to 7:1 (Table 4).

**Table 4:** Viricidal effect of HOCl solution on MHV-A59

Sample	HOCl	Mixing volume ratio (HOCl: viral suspension)				
		3:1	4:1	5:1	6:1	7:1
MHV-A59	Fresh	+	+	+	−	−
MHV-A59 with Saliva	Fresh	+	+	+	+	−

+ : Infectious. −: Not infectious

In dental practice, water is supplied through the DUWLs. Therefore, we investigated the disinfection effect of HOCl solution after passing through the DUWL. Before testing the disinfection effect, we confirmed the contamination level of the DUWL in our clinic. No heterotrophic bacteria were detected in one unit, while a maximum 25 colony-forming units (CFU)/ml of bacteria were found in the other unit. This was within the Centers for Disease Control and Prevention/Americans with Disabilities Act DUWL water quality guidelines of < 500 CFU/ml. The level of DUWL contamination was ignorable. The HOCl solution dispensed from the 3-way syringe after passing through the DUWL (DUWL-HOCl) required 8 times volume ratio to achieve sufficient antimicrobial effect. There was no difference in the amount of DUWL-HOCl required for bactericidal effect with or without saliva (Table 5).

**Table 5:** Bactericidal effect of HOCl solution passed through the DUWL

Bacteria	HOCl	Saliva	Mixing volume ratio (HOCl: bacterial suspension)						
			0.25: 1	0.5: 1	1: 1	2: 1	4: 1	8: 1	16: 1
*F. nucleatum*	DUWL-HOCl	Absent	+	+	+	+	+	−	−
*P. intermedia*	DUWL-HOCl	Absent	+	+	+	+	−	−	−
*P. micra*	DUWL-HOCl	Absent	+	+	+	+	−	−	−
*S. intermedius*	DUWL-HOCl	Absent	+	+	+	+	−	−	−
*F. nucleatum*	DUWL-HOCl	Present	+	+	+	+	+	−	−
*P. intermedia*	DUWL-HOCl	Present	+	+	+	+	+	−	−
*P. micra*	DUWL-HOCl	Present	+	+	+	+	+	−	−
*S. intermedius*	DUWL-HOCl	Present	+	+	+	+	+	−	−

DUWL-HOCl: HOCl passed through dental unit water line

+ : Growth was positive. −: No growth was observed

## Discussion

The efficacy of HOCl solution against pathogens is affected by many factors, including pH, chlorine concentration, and application method (duration of action, volume, and type of pathogen) [16, 30, 31]. It has been shown that increasing the volume, residual chlorine concentration, and treatment time of HOCl leads to improved microbicidal activity [32, 33]. In this study, we focused on the impact of usage, residual chlorine content, organic matter, supply route, and storage on the microbicidal activity of HOCl solution. In addition, the influence of saliva (an organic matter-rich body fluid) and the DUWL, which are specific to dental treatment, was examined. The duration of action of HOCl was fixed within 30 s in all assays, taking into account the effect of atmospheric oxygen on the strict anaerobes. Therefore, the duration effect on the microbicidal effect was not considered in this study. MHV-A59 was used as a substitute for SARS-CoV-2 in this study, since a BSL-3 lab is required for handling SARS-CoV-2. The adequacy of MHV-A59 as a substitute for SARS-CoV-2 has been previously shown [34, 35]. The efficacy of HOCl solutions against various viruses [36–40], including SARS-CoV-2 [41], has been confirmed in previous studies, and thus resistance of SARS-CoV-2 to HOCl solution may not be a major concern. However, we needed to assess the inhibition of HOCl efficacy by saliva.

Under the condition of a medium concentration of HOCl (45–60 ppm) and 30 s of duration, the bactericidal effect of HOCl solution was dose-dependent and completely inactivated all pathogens at a minimum inhibitory volume ratio 6:1 to microbial suspensions in the absence of saliva. Regarding bacteria, Si and Pm, both Gram-positive species, required higher volumes of HOCl solution than Gram negative Fn and Pi.

Increasing the volume of medium-concentration HOCl solution led to more effective microbicidal activity than the use of small volumes of high-concentration solution. This result may be related to the acidity-dependent activity of HOCl. High concentrations of HOCl solution were achieved by multiple cycles of 3% hydrochloric acid electrolysis. As a result, high concentration HOCl solutions were more acidic. The medium concentration HOCl solutions (45–60 ppm total chlorine content) used in most of this study exhibit a pH of about 4, whereas high concentration solution (about 400 ppm total chlorine content) was highly acidic with a pH of 1–2 (data not shown). It is known that HOCl chlorine species, the most oxidizing chlorine species in HOCl solutions, drastically decreases along with acidity when the pH is less than 3.5 [42, 43], and its bactericidal effect decreases. Therefore, in the use of a HOCl solution, the acidity as well as the concentration should be taken into consideration.

The presence of organic matter-rich saliva in the oral cavity is a characteristic disruptive factor for the efficacy of HOCl solution as disinfectant because organic compounds such as proteins consume HOCl chlorine species rapidly by oxidation reactions [44]. Even in the presence of saliva, all tested microorganisms were inactivated within 30 s by using at least 8 times the volume of HOCl solution, although the minimum growth inhibition volume ratio depended on the type of pathogen. Here, let us consider this minimum inhibitory volume ratio of 8:1 in conjunction with the oral situation. Saliva contains an average of 1.0 × 10^8^ bacteria/ml [45], a lower concentration than the bacterial suspension used in this study. The mean volume of residual saliva is 0.77 ml (range 0.38–1.73 ml) in the mouth [46] and a comfortable mouthwash volume on average is 15.0 ml [47]. The volume ratio of this residual saliva to mouthwash was 1:20, well above the minimum inhibitory volume ratio of 8:1. Thus, using a 15 ml medium concentration of HOCl solution as a mouthwash to effectively disinfect the oral cavity within 30 s seems to be a feasible approach.

A characteristic feature of dental practice is that water is supplied through long DUWL. The reduction of effective chlorine species by passing through the DUWL may be affected by the contamination level in the line. The treatment of DUWLs in the University of Michigan Graduate Endodontics Clinic maintained a low level of contamination, so the effect of the passage through DUWL on the minimum inhibitory volume ratio was very small (Table 5). Continuous use of HOCl solution may secondarily prevent contamination of the DUWL at the same time, but the need for treatment to clean up DUWL, such as Shock treatment, would be a matter of consideration. Storage is also an important factor that can decrease the residual chlorine content [16, 44]. The minimum inhibitory volume ratio increased after one week of storage at room temperature as shown in Table 1. Therefore, HOCl solutions must be prepared on demand or utilized within the shortest storage period possible.

Our in vitro research demonstrates that the HOCl solution has properties suitable for disinfection. However, the total residual chlorine content alone is not an indicator of the disinfection effect of the HOCl solution; the acidity and the volume of HOCl solution used should be considered simultaneously for optimal conditions. In addition, clinical studies of HOCl solution, including evaluation of patient acceptance, usability, and validation of the microbicidal effect in actual treatments, are essential for its clinical application.

## Conclusion

The HOCl solution, used at optimal concentrations and amounts under experimental conditions with dental clinical considerations, completely inactivated the human oral pathogen and the SARS-CoV-2 surrogate virus in less than 30 s. Most likely, the use of HOCl solution will directly prevent the contamination of the aerosol and droplets. Although some issues remain for its clinical application, the use of HOCl solutions in the treatment water in dentistry and mouthwash could be a promising solution to the long-standing unsolved problem of reducing the risk of airborne infection in dental treatment.

## Data Availability

The datasets used and/or analyzed during the current study are available from the corresponding author on reasonable request.

## Abbreviations

COVID-19: Coronavirus disease of 2019
SARS-CoV-2: Severe acute respiratory syndrome coronavirus 2
DUWLs: Dental unit waterlines
HOCl: Hypochlorous acid
WHO: World Health Organization
Fn: *Fusobacterium nucleatum*
Pi: *Prevotella intermedia*
Si: *Streptococcus intermedius*
Pm: *Parvimonas micra*
TSA: Tryptone soy agar
OD: Optical density
CPE: Cytopathogenic effect
CFU: Colony for unit
